# Understanding the public’s decision-making about seasonal flu vaccination during a pandemic: Application of the precaution adoption process model

**DOI:** 10.1177/13591053241296650

**Published:** 2024-11-22

**Authors:** Carly Meyer, Vivi Antonopoulou, Louis Goffe, Aikaterini Grimani, Fiona Graham, Jan Lecouturier, Mei Yee Tang, Paul Chadwick, Falko F Sniehotta

**Affiliations:** 1 University College London, UK; 2 Newcastle University, UK; 3 University of Warwick, UK; 4 Heidelberg University, Germany

**Keywords:** health belief model, influenza, public health, respiratory infectious disease, theory of planned behaviour, vaccine hesitancy

## Abstract

Understanding the behavioural factors influencing flu vaccination is crucial for mitigating seasonal infection outbreaks. This study utilised the Precaution Adoption Process Model (PAPM) to examine the public’s decision-making about seasonal flu vaccination through an online cross-sectional survey with 2004 participants in England, UK. Results showed varying stages of decision-making: 7% in Stage 2 (unengaged), 10% in Stage 3 (undecided), 7% in Stage 4 (decided not to vaccinate), 39% in Stage 5 (decided to vaccinate) and 38% in Stage 6 (vaccinated). Regression modelling revealed factors common across stages and unique to certain stages, such as flu vaccination history distinguishing those who received the vaccine. Vaccine knowledge (Stage 2), perceived benefits (Stage 4), perceived control and fear of needles (Stage 5) were uniquely associated with specific PAPM stages. The study discusses policy implications for integrating these findings to improve flu vaccination uptake, highlighting the importance of tailored strategies based on decision-making stages.

## Introduction

Annual vaccination against seasonal influenza (flu) is recommended by the World Health Organisation (WHO) for people 65 years and older and for people with chronic health conditions as they are more susceptible to infection-related complications (Blank et al., 2012; Wheelock et al., 2013; World Health Organization n.d). The WHO emphasised the need to maintain optimal flu care during the COVID-19 pandemic when medical resources and attention were increasingly being directed towards COVID-19 (McCauley et al., 2022). In England, during the COVID-19 pandemic, eligibility for the seasonal flu vaccination programme for the winter of 2020–2021 was extended to include adults aged 50–64 years old (previously 65 years and over) and household members of individuals with underlying health conditions who were identified by the National Health System (NHS) as at high risk of complications from COVID-19. Thus, approximately 32.4 million people were eligible for a free flu vaccine in England, representing about half the population (Nuffield Trust, 2022, March 31). Between 2015 and 2019, flu vaccine uptake had decreased with coverage rates below current national ambitions of ≥75% in all clinical risk groups. However, the 2020–2021 season was the first time the WHO target was met since winter 2005–2006 with uptake in people aged 65 years and over reaching 80.9% (Nuffield Trust, 2022, March 31). For the 2021–2022 season, when both flu and COVID-19 were circulating, the government emphasised the importance of being vaccinated against both viruses and increased targets for England’s flu programme, aiming for 85% coverage among over 65s and 75% coverage among eligible under 65s (Iacobucci, 2021).

In relation to flu vaccination more specifically, it was proposed that the COVID-19 pandemic could potentially create an opportunity for an increase in demand for flu vaccines, particularly for high priority and vulnerable groups, given that the pandemic had elevated public awareness and concern about health (Jaklevic, 2020). Therefore, this was a unique opportunity amidst a pandemic to understand flu vaccine behaviour and how COVID-19 might have impacted vaccine uptake through its influence on common determinants of vaccine behaviour.

### Factors influencing flu vaccine uptake

Previous research highlights that vaccine acceptance is a complex decision-making process influenced by a wide range of psychological, health, and socio-demographic factors (Butter et al., 2022; Goffe et al., 2024). Seven psychological and behavioural factors have been most frequently and consistently identified as being significantly associated with flu vaccine intention and uptake (Bish et al., 2011; Lorenc et al., 2017; Maltezou et al., 2010; Schmid et al., 2017). These include: perceived susceptibility to disease (not perceiving oneself as vulnerable to the disease) (Han et al., 2016), perceived severity of the disease and perceived benefits of vaccination, attitude towards flu vaccination, vaccine-related side effects (Nagata et al., 2013), social norms (i.e. the perceived pressure or expectation that significant others would want them to get the vaccine) (Quinn et al., 2017a), anticipating feelings of regret (i.e. the impact of missing a vaccination opportunity) (Gallagher and Povey, 2006), and previous flu vaccinations (Gidengil et al., 2012; Sherman et al., 2021a). These factors represent key constructs in theories of behaviour change such as the Theory of Planned Behaviour (TPB) (Ajzen, 1991) and Health Belief Model (HBM) (Rosenstock, 1974; Rosenstock et al., 1988). Contextual factors include physical barriers such as access, vaccine availability, and previous vaccination experiences (Boey et al., 2018; Gallant et al., 2023). Importantly, socio-demographic factors such as age (Abbas et al., 2018), gender (Mo and Lau, 2015a), educational level (Takayama et al., 2012) and ethnicity (Bish et al., 2011; Quinn et al., 2017b) have also been associated with flu vaccine intention and uptake.

### Theoretical framework

The WHO Scientific Advisory Group for Emergencies (SAGE) Working Group on Vaccine Hesitancy defines vaccine hesitancy as a continuum ranging from complete refusal of all vaccines, delay or refusal of some vaccines but acceptance of others, to acceptance of all vaccines and defines it as a delay in acceptance or refusal of vaccination despite availability of vaccination services (Larson et al., 2014; MacDonald, 2015). Thus, people may go through various stages of decision-making before having a vaccine which can be best captured using a stage of change-based framework. The Precaution Adoption Process Model (PAPM) explains how individuals make decisions to adopt new precautions or cease risky behaviours, a process that involves conscious, deliberate steps (stages) rather than automatic habits, like for example, adopting physical activity habits (Weinstein et al., 2020). Stages are theoretical constructs, with each representing an ‘ideal’ prototype, that not all individuals align with perfectly. Unlike theories that view behaviour as a binary decision to act or not act, stage theories, like the PAPM, typically assume that people move through a series of different stages before taking action and that predictions about behaviour require different factors to be in place for each stage. However, the progression is not always straightforward and can vary, with individuals spending different amounts of time in each stage, and some may decide not to act at all. This variability reflects the complex nature of decision-making and behaviour change (Weinstein et al., 2020). The PAPM postulates that adopting preventive health behaviour involves six distinct sequential stages of decision-making: (1) being unaware of the health behaviour; (2) being unengaged in the decision; (3) being undecided about whether to act or not; 4() deciding not to act; (5) deciding to act (intending) and (6) acting (having the vaccination) (Weinstein et al., 2008; Weinstein and Sandman, 1992). The PAPM’s detailed breakdown – ranging from being unaware to taking action or deciding not to – offers a nuanced understanding of flu vaccination adoption. This stage-based approach enables a more precise identification of barriers and facilitators at each step. However, the PAPM complements other models, as it does not provide a predetermined set of variables for distinguishing between stages. Instead, individuals’ progress through the PAPM stages is based on their attitudes and beliefs (Elliott et al., 2007). To enhance this understanding, constructs from other models, such as perceived benefits, control, susceptibility, and disease severity from the HBM and the extended TPB, can be integrated to better understand how individuals transition between stages. Previously, this model has been applied to human papillomavirus (HPV) vaccination decision-making (Shapiro et al., 2018; Waller et al., 2020) and more recently to COVID-19 vaccination (Meyer et al., 2023), but not, to our knowledge, to flu vaccination. An understanding of factors associated with each stage of flu vaccine decision-making can inform the design of more tailored interventions at different stages to increase flu vaccination rates ahead of future flu seasons and pandemics. This targeted approach can enhance the effectiveness of public health campaigns aimed at promoting flu vaccination. Importantly, the PAPM considers the dynamic nature of behaviour change by acknowledging that individuals may progress through different stages over time. This sequential perspective is crucial for understanding the evolving nature of flu vaccine decision-making, especially in response to changing public health contexts.

#### Current study

The aim of the present study was twofold: first, to use the PAPM to profile cross-sectionally the public’s decision-making about having the seasonal flu vaccine; and second, to examine associations between each PAPM stage and attitudes and beliefs about the vaccine, personal health characteristics, and socio-demographic characteristics.

## Method

### Ethics

Ethics approval was obtained from Newcastle University Ethics Committee (Reference: 13754/2020). Informed consent was obtained from all participants involved in the study.

### Design

This was an exploratory cross-sectional study about seasonal flu vaccination, conducted as part of a larger study examining factors influencing vaccine decision making for both the COVID-19 booster vaccine (Meyer et al., 2023) and seasonal flu vaccine in the second year of the Covid-19 pandemic. We administered an online, cross-sectional survey to people living in England approximately 4 weeks after the COVID-19 booster vaccine programme commenced and the seasonal flu vaccine programme was underway (October 11–20, 2021) (Department of Health & Social Care, 2021; Public Health England, 2021).

### Participants

Participant inclusion criteria aligned with age-based vaccine eligibility criteria at the time we conducted the study: participants needed to live in England, be 50 years of age or older, and have previously completed the primary course of the COVID-19 vaccine (i.e. received two doses). Quotas were used to ensure a nationally (English) representative sample in terms of gender and region. Participants were recruited using Qualtrics panel recruitment. Participants who completed the survey were incentivised through Qualtrics automatic rewards-based programme as reimbursement for their time.

### Materials

The online questionnaire was adapted from one previously used to investigate seasonal flu vaccine intention (Antonopoulou et al., 2022) and was informed by the PAPM (Weinstein and Sandman, 1992), the extended Theory of Planned Behaviour (Ajzen, 1991) and the Health Belief Model (Rosenstock, 1974). Additional questions asked about past flu vaccine experience and personal health and socio-demographic characteristics. All questions are available in Supplemental File 2.

### PAPM stage for seasonal flu vaccination

Participants were asked ‘*Which of the following best describes your thoughts about having a seasonal flu vaccine this year?*’ and were asked to select one of six options that related to Stages 2–6 of the PAPM (see Supplemental File 2). Options pertaining to Stages 1 (unaware) and 7 (maintenance) were omitted because the seasonal flu vaccination programme was well established and therefore, it was highly unlikely that participants would be unaware of the COVID-19 and the flu vaccination programmes. Similarly, Stage 7 (maintenance) was not included as although the behaviour can be repeated annually, it is not a behaviour that needs to be maintained. Omitting Stage 7 streamlined our survey to concentrate on stages characterised by more active decision-making. Overall, our targeted approach was intended to ensure that our research captured the more pronounced elements of flu vaccine decision-making without unnecessary redundancy on stages where awareness is reasonably assumed (Stage 1) or maintenance is inherently straightforward (Stage 7).

### Previous vaccine experience

Questions focussed on individuals’ previous flu vaccination behaviour (2 items) and if applicable, their overall experience of receiving their previous flu vaccine (4 items). All items were measured on a 5-point Likert scale, with higher scores representing higher frequency or more positive experiences. A mean subscale score was computed for previous flu vaccine experience as the scale was found to have good internal consistency (Cronbach’s alpha = 0.77).

### Beliefs and attitudes about seasonal flu vaccination

Questions informed by the extended TPB pertained to vaccine attitudes (2 items), vaccine subjective norms (4 items), vaccine perceived control (1 item), and anticipated regret (3 items). Questions informed by the HBM represented perceived severity (1 item), perceived susceptibility (2 items), perceived benefits (5 items), and perceived vaccine safety (2 items). Additional questions asked about knowledge of vaccine safety and effectiveness (3 items), trust in Government (1 item; adapted from (Meyer, 1988)), and fears of having a vaccine (4 items; (Myers and Goodwin, 2011)). Survey questions were derived from published studies that also examined the psychological determinants of vaccine intention (Hamilton et al., 2021; Myers and Goodwin, 2011; Sherman et al., 2021b; Ziarnowski et al., 2009). All items (except ‘fears of having a vaccine’) were measured on a 5-point Likert scale, with higher scores representing higher levels of agreement/more anticipated regret. All constructs aside from ‘perceived susceptibility’ had good internal consistency (Cronbach’s alpha 0.69–0.96) and were represented as a single mean score.

### Personal health characteristics and socio-demographic characteristics

Participants were asked to indicate their age, gender, region, ethnicity, education status, employment status, keyworker status, household income, general health, and whether or not they were asked to shield from COVID-19 during the pandemic.

### Patient and public involvement

The survey was reviewed twice by our Unit’s dedicated Patient and Public Involvement (PPI) team (*N* = 6, five aged 50 and over, consistent with target population) to check the relevance and understandability of survey items and to ensure the online survey was easy to use.

### Data analysis

STATA (version 16) was used to carry out all analyses. Univariate associations between PAPM stage and all potential variables were first examined using univariate, multinomial logistic regression models and significant variables (*p* < 0.10) were considered for inclusion in the multivariate model. Multicollinearity was checked by inspecting variation inflation factor (VIF) values; all were less than 3.0 indicating minimal evidence of multicollinearity. Pairwise correlations were computed between all continuous variables; the variable ‘vaccine attitude’ was dropped because it was highly correlated with ‘perceived safety’ (*r* = 0.719) and ‘subjective norms’ (*r* = 701). All other correlations were less than 0.7 (see Supplemental File 3). Two variables were excluded from multivariate modelling due to high numbers of missing data, including overall experiences with past flu vaccination (*n* = 1460; 27% missing data) and fear of vaccine side effects lasting longer than 2 days (*N* = 1563; 22% missing data). A multivariate, multinomial logistic regression model was fitted to the data to represent factors associated with each stage of the PAPM model; participants in PAPM Stage 6 (vaccinated) served as the reference group and was compared to participants in Stage 2 (unengaged), Stage 3 (undecided), Stage 4 (decided not to vaccinate), and Stage 5 (decided to vaccinate). Backward stepwise regression was used to simplify model selection by identifying the most relevant predictors in a data-driven way, which aligns well with the exploratory nature of our analysis. A conservative *p*-value of 0.10 was used for factor inclusion; however, statistical significance was inferred by p-values <0.05. The relative risk ratios (RRR) and 95% confidence intervals are reported; RRRs < 1 indicate a negative association with PAPM stage and RRRs > 1 indicate a positive association. Goodness of fit is represented by the pseudo *r*^2^ value.

## Results

Overall, 2004 people participated in the study. On average, participants were 64 years of age (SD = 8.45) and 51% of the sample were female. We had representation across the nine regions of England and most reported they were white British (92%). About one-third of the sample were currently employed in part-time or full-time work and about half the sample reported a household income less than £30,000. (Supplemental File 1 presents a summary of participants’ demographic information).

Of the 2004 participants, 132 (7%) were in Stage 2 (unengaged), 199 (10%) were in Stage 3 (undecided), 131 (7%) were in Stage 4 (decided not to vaccinate), 790 (39%) were in Stage 5 (decided to vaccinate), and 752 (38%) were in Stage 6 (vaccinated). Table 1 presents socio-demographic, socio-economic and health information by PAPM stage; and Table 2 presents participants’ attitudes and beliefs towards seasonal flu vaccination.

**Table 1.** Socio-demographic, socio-economic, and health information for participants, by PAPM stage of decision-making (*N* = 2004).

	Unengaged		Undecided		Decided not to vaccinate		Decided to vaccinate		Vaccinated		Univariate analyses^a^	
Variable	(*n* = 132)		(*n* = 199)		(*n* = 131)		(*n* = 790)		(*n* = 752)		*N*	pseudo *r*^2^
Age – *M* (SD)	58.37	(6.52)	60.55	(7.57)	62.49	(8.29)	63.32	(8.33)	65.83	(8.40)	2004	0.026***
Gender – *N* (%)											2004	0.001
Female or Other	67	(6.55)	90	(8.80)	67	(6.55)	408	(39.88)	391	(38.22)		
Male	65	(6.63)	109	(11.11)	64	(6.52)	382	(38.94)	361	(36.80)		
Region – *N* (%)											2004	0.010***
London	22	(7.41)	33	(11.11)	31	(10.44)	91	(30.64)	120	(40.40)		
South East	27	(8.44)	34	(10.63)	11	(3.44)	134	(41.88)	114	(35.63)		
South West	5	(2.50)	18	(9.00)	9	(4.50)	87	(43.50)	81	(40.50)		
West Midlands	15	(6.73)	22	(9.87)	18	(8.07)	96	(43.05)	72	(32.29)		
North West	16	(6.18)	19	(7.34)	18	(6.95)	95	(36.68)	111	(42.86)		
North East	10	(9.62)	9	(8.65)	3	(2.88)	36	(34.62)	46	(44.23)		
Yorkshire and the Humber	13	(6.50)	29	(14.50)	10	(5.00)	81	(40.50)	67	(33.50)		
East Midlands	12	(6.63)	14	(7.73)	12	(6.63)	77	(42.54)	66	(36.46)		
East of England	12	(5.45)	21	(9.55)	19	(8.64)	93	(42.27)	75	(34.09)		
Ethnicity – *N* (%)											1998	0.004***
White British	110	(5.97)	173	(9.39)	117	(6.35)	741	(40.21)	702	(38.09)		
Not white British	20	(12.90)	25	(16.13)	14	(9.03)	48	(30.97)	48	(30.97)		
Education status – *N* (%)											1996	0.002
No formal qualification	8	(5.41)	11	(7.43)	12	(8.11)	59	(39.86)	58	(39.19)		
High school qualification	70	(7.93)	89	(10.08)	65	(7.36)	342	(38.73)	317	(35.90)		
University diploma/degree	25	(4.88)	47	(9.18)	30	(5.86)	209	(40.82)	201	(39.26)		
Other qualification	27	(5.96)	51	(11.26)	24	(5.30)	177	(39.07)	174	(38.41)		
Employment status – *N* (%)											1996	0.011***
Employed	71	(9.45)	108	(14.38)	50	(6.66)	295	(39.28)	227	(30.23)		
Not employed	60	(4.82)	90	(7.23)	80	(6.43)	492	(39.52)	523	(42.01)		
Key worker status – *N* (%)											2004	0.004***
Yes	33	(10.68)	44	(14.24)	12	(3.88)	122	(39.48)	98	(31.72)		
No	99	(5.84)	155	(9.14)	119	(7.02)	668	(39.41)	654	(38.58)		
Health or social care worker – *N* (%)											2004	<0.001
Yes	6	(6.74)	10	(11.24)	3	(3.37)	38	(42.70)	32	(35.96)		
No	126	(6.58)	189	(9.87)	128	(6.68)	752	(39.27)	720	(37.60)		
Household income – *N* (%)											2004	0.002
Less than £30,000	53	(6.22)	81	(9.51)	47	(5.52)	350	(41.08)	321	(37.68)		
More than £30,000	74	(7.49)	100	(10.12)	72	(7.29)	377	(38.16)	365	(36.94)		
Prefer not to say	5	(3.05)	18	(10.98)	12	(7.32)	63	(38.41)	66	(40.24)		
General health – *M* (SD)	2.32	(0.95)	2.34	(0.85)	2.32	(0.96)	2.46	(0.86)	2.49	(0.84)	1999	0.002**
Asked to shield – *N* (%)											1996	0.007***
Yes	15	(3.78)	26	(6.55)	11	(2.77)	155	(39.04)	190	(47.86)		
No	116	(7.25)	173	(10.82)	117	(7.32)	633	(39.59)	560	(35.02)		
Previous COVID-19 infection – *N* (%)											2002	<0.001
Yes	18	(8.33)	27	(12.50)	15	(6.94)	87	(40.28)	69	(31.94)		
No	114	(6.38)	172	(9.63)	116	(6.49)	703	(39.36)	681	(38.13)		

PAPM: precaution adoption process model.

a Based on univariate multinomial regression model, with reference category ‘Stage 6: Vaccinated’.

* *p* < 0.10. **p < 0.05. ***p < 0.01.

**Table 2.** Experiences, attitudes, and beliefs about seasonal flu vaccination, by PAPM stage of decision-making (*N* = 2004).

	Unengaged		Undecided		Decided not to vaccinate		Decided to vaccinate		Vaccinated		Univariate analyses^a^	
Variable	(*n* = 132)		(*n* = 199)		(*n* = 131)		(*n* = 790)		(*n* = 752)		*N*	pseudo *r*^2^
Previous flu vaccine experience												
Overall experience – *M* (SD)^b^	4.31	(0.88)	4.23	(0.81)	3.11	(0.69)	4.73	(0.46)	4.74	(0.47)	1460	0.031***
Flu vaccine frequency – *M* (SD)	1.90	(1.30)	2.16	(1.43)	1.27	(0.57)	4.01	(1.49)	4.68	(0.82)	2004	0.188***
Psychological constructs												
Perceived severity – *M* (SD)	3.22	(0.96)	3.28	(0.91)	2.63	(1.02)	3.75	(0.95)	3.89	(0.92)	2004	0.045***
Perceived susceptibility: high risk of catching flu – *M* (SD)	2.73	(1.08)	2.82	(0.96)	2.17	(0.97)	3.32	(1.07)	3.41	(1.08)	2004	0.040***
Perceived susceptibility: immune system strong enough – M (SD)	3.53	(0.89)	3.34	(0.77)	3.66	(0.96)	3.10	(1.11)	3.27	(1.18)	2004	0.008***
Perceived benefits – *M* (SD)	3.73	(0.70)	3.80	(0.59)	3.05	(0.83)	4.25	(0.60)	4.32	(0.58)	2004	0.086***
Perceived safety – *M* (SD)	3.64	(0.84)	3.56	(0.76)	3.02	(0.88)	4.26	(0.72)	4.47	(0.63)	2004	0.100***
Vaccine attitudes – *M* (SD)	3.74	(0.75)	3.84	(0.65)	2.82	(0.82)	4.55	(0.60)	4.73	(0.44)	2004	0.170***
Subjective norms – *M* (SD)	3.37	(0.77)	3.49	(0.63)	2.86	(0.73)	4.18	(0.71)	4.36	(0.65)	1996	0.120***
Perceived control – *M* (SD)	3.99	(0.73)	4.05	(0.74)	4.16	(0.87)	4.51	(0.69)	4.70	(0.54)	2004	0.044***
Anticipated regret – *M* (SD)	3.76	(1.46)	4.07	(1.06)	3.12	(1.34)	4.57	(0.99)	4.69	(0.83)	2004	0.048***
Vaccine knowledge – *M* (SD)	3.45	(0.90)	3.63	(0.74)	3.40	(0.84)	4.32	(0.64)	4.47	(0.61)	2004	0.092***
Trust – *M* (SD)	3.47	(1.01)	3.62	(0.82)	3.21	(0.97)	4.17	(0.79)	4.33	(0.81)	2004	0.055***
Vaccination fears												
Side effects last ≤2 days – *N* (%)											1563	0.021***
Yes	60	(4.18)	105	(7.31)	44	(3.06)	609	(42.38)	619	(43.08)		
No	3	(2.38)	24	(19.05)	27	(21.43)	41	(32.54)	31	(24.60)		
More side effects from COVID-19 vaccine versus flu vaccine – *N* (%)											1015	0.002
Yes	35	(5.27)	68	(10.24)	36	(5.42)	267	(40.21)	258	(38.86)		
No	15	(4.27)	23	(6.55)	26	(7.41)	143	(40.74)	144	(41.03)		
All vaccines cause side-effects – *N* (%)											1773	<0.001
Yes	104	(6.37)	157	(9.62)	106	(6.50)	637	(39.03)	628	(38.48)		
No	7	(4.96)	11	(7.80)	8	(5.67)	62	(43.97)	53	(37.59)		
Fear of needles – *N* (%)											1970	0.003***
Yes	30	(9.84)	39	(12.79)	23	(7.54)	100	(32.79)	113	(37.05)		
No	97	(5.83)	157	(9.43)	103	(6.19)	680	(40.84)	628	(37.72)		

PAPM: precaution adoption process model.

a Based on univariate multinomial regression model, with reference category ‘Stage 6: Vaccinated’.

b Only asked to 1460 people who have had the flu vaccine previously.

* *p* < 0.10. **p < 0.05. ***p < 0.01.

Twelve factors relating to participants’ attitudes and beliefs towards seasonal flu vaccination, their past flu vaccine behaviour, and personal demographic characteristics were associated with PAPM decision-making stage in the final multivariate model (*n* = 1946, log likelihood = −1835.69, LR χ^2^ (56) = 1433.54, pseudo *r*^2^ = 0.281, *p* < 0.0001) (see Table 3). Relative to people who had already received the seasonal flu vaccine, being *unengaged* was associated with younger age, a history of less frequent flu vaccination behaviour, weaker subjective norms, less anticipated regret, stronger beliefs their immune system would protect against seasonal flu, and less perceived vaccine knowledge. Being *undecided* was associated with a history of less frequent flu vaccine behaviour, weaker subjective norms, less anticipated regret, less perceived safety, and living in Yorkshire and the Humber (vs London). Making the *decision not to have the flu vaccine* was associated with a history of less frequent flu vaccine behaviour, weaker subjective norms, less anticipated regret, stronger beliefs that their immune system would protect against seasonal flu, less perceived safety, fewer perceived benefits of vaccination, and less likelihood of living in the North East (vs London). Lastly, making the *decision to have the flu vaccine* was associated with younger age, a history of less frequent flu vaccine behaviour, weaker beliefs that their immune system would be strong enough to protect against flu, less perceived safety, less perceived control, and less likelihood of being fearful of needles.

**Table 3.** Multivariate, multinomial backward stepwise logistic regression model for different stages of decision making about getting the seasonal flu vaccine, represented by PAPM stage, displaying adjusted relative risk ratios and 95% CIs (*n* = 1946).

	Vaccinated (*n* = 737) vs …							
	Unengaged (*n* = 121)		Undecided (*n* = 194)		Decided not to vaccinate (*n* = 123)		Decided to vaccinate (*n* = 771)	
Variable	aRRR	95% CI	aRRR	95% CI	aRRR	95% CI	aRRR	95% CI
Age	0.958*	[0.925,0.992]	0.997	[0.971,1.025]	1.037	[0.997,1.079]	0.983*	[0.969,0.996]
White British (vs not)	0.478	[0.227,1.006]	0.595	[0.304,1.167]	0.753	[0.277,2.045]	1.388	[0.881,2.187]
Flu vaccine frequency	0.355***	[0.295,0.426]	0.374***	[0.322,0.434]	0.166***	[0.111,0.246]	0.641***	[0.577,0.712]
Subjective norms	0.533**	[0.352,0.807]	0.612**	[0.428,0.873]	0.284***	[0.169,0.477]	1.009	[0.820,1.241]
Anticipated regret	0.723**	[0.581,0.899]	0.811*	[0.666,0.986]	0.592***	[0.455,0.770]	0.898	[0.791,1.019]
Perceived susceptibility: immune system strong enough	1.545**	[1.163,2.052]	1.249	[0.997,1.564]	1.761**	[1.224,2.535]	0.880**	[0.800,0.969]
Perceived safety	0.681	[0.450,1.030]	0.459***	[0.325,0.648]	0.239***	[0.151,0.378]	0.760*	[0.611,0.944]
Perceived benefits	1.057	[0.681,1.641]	1.036	[0.704,1.523]	0.533*	[0.319,0.893]	1.250	[0.995,1.571]
Perceived control	0.828	[0.555,1.236]	0.790	[0.555,1.125]	1.483	[0.924,2.379]	0.739*	[0.582,0.936]
Fear of needles (vs not)	1.064	[0.587,1.930]	0.896	[0.535,1.500]	0.758	[0.358,1.606]	0.674*	[0.493,0.921]
Vaccine knowledge	0.438***	[0.282,0.681]	0.681	[0.459,1.010]	0.889	[0.540,1.463]	1.153	[0.881,1.508]
Region								
London	Reference		Reference		Reference		Reference	
North West	1.507	[0.743,3.059]	0.876	[0.464,1.654]	2.119	[0.862,5.210]	0.740	[0.539,1.015]
North East	1.322	[0.494,3.542]	0.626	[0.235,1.668]	0.104*	[0.0151,0.725]	0.734	[0.457,1.180]
Yorkshire & the Humber	1.369	[0.608,3.081]	2.150*	[1.167,3.962]	0.753	[0.240,2.363]	1.172	[0.811,1.692]

PAPM: precaution adoption process model.

* *p* < 0.05. **p < 0.01. ***p < 0.001.

## Discussion

The findings of this study indicated that a high proportion (77%) of our sample were favourable to the flu vaccine by either having been vaccinated or having made the decision to be vaccinated. Of the remaining individuals in our sample, only a small proportion had decided not to vaccinate (7%). However, it is important to note that all participants were eligible to receive a flu vaccine free of charge at the time these data were collected. During the period of the study (October 2021), flu vaccines in England were widely accessible at GP practices and local pharmacies and special provisions were made for at-risk groups to receive their vaccines at care homes (PHE, 2020). Although there were some commonalities across PAPM decision-making stages, by using a nuanced approach to understanding flu vaccine behaviour, we were also able to elucidate factors that differentiated people at different stages of decision-making about having the seasonal flu vaccine.

Consistent with previous literature (Frew et al., 2012; Kan and Zhang, 2018; Mayo and Cobler, 2004; Mo and Lau, 2015; Sherman et al., 2021a; Wheelock et al., 2013), people who were previously vaccinated were in later stages of decision-making, although the effect of frequency of vaccination was stronger for people who had decided they would not have the flu vaccine (i.e. for those being in an earlier stage of decision-making).

Four factors were found to be unique to a particular stage of decision-making, including vaccine knowledge, perceived benefits of vaccination, perceived control, and fear of needles. Less knowledge about flu vaccine safety and effectiveness was a significant correlate of being ‘unengaged’, compared to people who were already vaccinated, suggesting that education about vaccine safety and effectiveness may play an important role in engaging a person in the decision-making process but alone would not be sufficient to increase vaccine uptake. Perceiving fewer benefits of flu vaccination was a significant correlate of deciding not to have the vaccine. This is despite the fact that all participants in our study cohort had received two doses of the COVID-19 vaccine and therefore were assumed unlikely to hold anti-vaccination views. In general, people who have decided against vaccination are likely to be more resistant to change (Larson et al., 2011); however, it seems crucial that they understand how they might benefit from flu vaccination if we are trying to increase flu vaccine uptake in this population group. People who had decided to have the vaccine reported less perceived control over vaccination relative to people who were already vaccinated indicating this might be an important factor to address to decrease the intention to behaviour gap. Fear of needles was also negatively correlated with deciding to vaccinate but given that the proportion of people who feared needles was greater among those who had already received the vaccine, this finding suggests that public health initiatives aimed at translating high vaccine intention into action do not need to target a fear of needles.

Overlapping factors that were significant across more than one PAPM stage included subjective norms, anticipated regret, perceived safety, beliefs about the immune system being strong enough, and age. Weaker subjective norms and less anticipated regret were significant correlates of being ‘unengaged’, ‘undecided’, or ‘decided not to vaccinate’, but not ‘decided to vaccinate’, indicating these beliefs stabilise once the decision to have the vaccination has been made. However, because our study employed a cross-sectional design, we are limited in our ability to assess changes over time. An alternative explanation is that individuals with weak subjective norms or low anticipated regret may represent a distinct group who might choose not to vaccinate in the future. In contrast, greater safety concerns about the flu vaccine appeared to be associated with all earlier stages of decision-making (with the exception of people who were ‘unengaged’), emphasising the need to alleviate any concerns people might have about the safety of the flu vaccine (including any potential side effects) to progress individuals towards making the decision to vaccinate and then converting that to action. Longitudinal research is needed to verify this finding.

Previous literature has highlighted the role of modifiable social and psychological variables in explaining vaccination behaviour (Nagata et al., 2013; Roller-Wirnsberger et al., 2021; Telford and Rogers, 2003; Wheelock et al., 2017; Yeung et al., 2016). Together, these findings suggest that policymakers could increase flu uptake rates by leveraging social connections and adopting community-led strength-based approaches to normalise vaccination behaviour and highlight the possible negative consequences of choosing not to have the vaccine, as well as reassure people about flu vaccine safety. One example is the ‘community champions’ initiative, where community members volunteer to promote health and wellbeing by providing reliable information and organising vaccination events alongside social and cultural activities (Gilburt et al., 2024).

Relative to people who had already received the flu vaccine, people who were unengaged or who had decided not to have the flu vaccine had stronger beliefs that their immune system would be strong enough to protect against seasonal flu. This finding is consistent with other studies that found that stronger beliefs about immune system capability were associated with lower vaccine uptake (Domnich et al., 2020; Gidengil et al., 2012; Hamilton et al., 2021; Telford and Rogers, 2003). In contrast, people who had decided to have the vaccine reported significantly weaker beliefs compared to people who were already vaccinated. This latter finding might reflect the fact that people who had already received the vaccine might have felt well protected against the flu due to vaccine-induced immunity. Nonetheless, it appears that people in the earlier stages of decision-making would benefit from knowing more about how they too might be susceptible to catching the flu and the impact that might have on their health and day-to-day life.

Age was the only statistically significant socio-demographic influence on flu vaccine behaviour; younger age was found to be a significant correlate of being unengaged or making the decision to have the vaccine, but not yet acting on it. This is consistent with previous literature which has highlighted that older age is associated with greater uptake of the flu vaccine (Marín-Hernández et al., 2021; Nagata et al., 2013; Sammon et al., 2012) and needs to be taken into consideration when designing interventions that target particular segments of the population. However, the limited demographic characteristics, that is, ethnic representation, of the sample does place constraints on the generalisability of the findings to the wider population of 50+ year olds. Therefore, caution is needed regarding the potential application of these findings to public health vaccination efforts targeted at the broader 50+ population and there is a need for further research to assess the applicability of these findings to a more diverse and representative population of older adults. Specific regions in England were also found to correlate with specific PAPM stages and these findings could be linked to local and regional social factors, although this may be a statistical artefact and should be interpreted with caution.

### Comparison with COVID-19 vaccination

When examining factors associated with COVID-19 booster vaccine decision-making also using the PAPM model for another study focussing on COVID-19 booster vaccine with the same cohort of participants (Meyer et al., 2023) knowledge about vaccine safety and effectiveness and perceived susceptibility (i.e. perception that the immune system was strong enough to protect against COVID-19) were correlated with being ‘unengaged’ or ‘undecided’ about having a booster vaccine. When compared to the present study examining decision-making about the seasonal flu vaccine, it is clear that perceived susceptibility is an important factor for both vaccines; however, knowledge about vaccine safety and effectiveness appears less important once engaged in the decision-making process.

In contrast to the flu findings, only people who had not yet engaged in the decision-making process reported weaker subjective norms for the COVID-19 booster vaccine (Meyer et al., 2023), whereas weaker subjective norms were reported across all PAPM stages for the flu vaccine except for those who had decided to have the flu vaccine. Thus, creating societal expectations around vaccination appears warranted for individuals at all PAPM stages in relation to flu vaccination but only for engaging people in the decision-making process in relation to COVID-19 booster vaccination. A potential explanation for these differences may be that while the urgency and heightened risk perception surrounding COVID-19 led to rapid vaccine uptake in some populations, flu vaccination might involve different decision-making processes due to the lower perceived severity and urgency. This could help explain why individuals move through the stages of the PAPM differently for these two vaccines. Another possible explanation is that these differences reflect the distinct communication strategies used during the two vaccination campaigns, as well as the differing public health priorities during the recent pandemic, when health concerns about COVID-19 were more prominently prioritised. Interestingly, socio-demographic factors appeared to account for more variance in vaccine decision-making for COVID-19 relative to seasonal flu. Where being unengaged in the decision to have a COVID-19 vaccine was associated with being employed, not having a formal educational qualification, and having a household income less than £30,000; and being undecided was associated with being an ethnicity other than White (Meyer et al., 2023); younger age was the only significant corelate of being ‘unengaged’ or ‘deciding to vaccinate’ in relation to flu vaccination.

### Policy implications

As the decline in antibodies against the influenza viruses over time necessitates annual revaccination, particularly for older adults and other at-risk groups, the need for successful public health campaigns every year is evident. The current findings can be useful at a strategic level for targeting groups identified as being at different levels of decision-making by highlighting which constructs are useful or which ones are under-utilised in the promotion of flu vaccine uptake. Previous studies conducted on people’s preferences for vaccination suggest that a more nuanced approach to the selection of persuasive campaign elements is the most effective strategy (Ling et al., 2019; Roller-Wirnsberger et al., 2021; Su et al., 2021). Our findings indicate that targeting vaccine knowledge may be most crucial among those not engaged in the decision-making process, whereas providing reassurance regarding flu vaccine safety and highlighting the benefits of vaccination may be most crucial among people who are undecided or who have decided not to vaccinate. Addressing social norms, anticipated regret, and perceived susceptibility could promote vaccine uptake among all people who are at earlier stages of decision-making, although could be most useful if targeting people who are unengaged or who have decided not to vaccinate. Importantly, however, flu vaccine frequency is negatively correlated with all earlier stages of decision-making indicating the need to focus attention on people who have never or rarely had the flu vaccine previously.

The application of the PAPM in this study provides a dynamic, stage-based understanding of vaccination decision-making, which contrasts with single-point theoretical models. By examining decision-making as a process with distinct stages, even if the boundaries between the stages aren’t always clear, this approach enables the identification of common barriers faced by people in the same stage and can help improve intervention effectiveness by designing targeted interventions. Future research could build on this methodological approach by applying the PAPM to other health behaviours or vaccination campaigns, allowing for a more comprehensive understanding of how decision-making processes evolve over time and in response to different public health communications.

### Strengths and limitations

Some limitations of this study are important to consider when interpreting these findings. Firstly, this was an online cross-sectional self-report study and as such, while it facilitates timely data collection, this method may introduce biases in the data in terms of sampling representativeness (requiring internet access, being registered with the survey platform) and it reflects vaccination attitudes and behaviour at the specific time point of data collection. Therefore, future research with longitudinal designs would be crucial to validate and expand upon our findings. Longitudinal studies would allow for a deeper understanding of how temporal factors influence behaviour over time and can help confirm the robustness of the stage-based models such as the PAPM. Secondly, our sample was a predominantly white British sample (92% vs ca. 80% in the population of England based on the latest census (ONS 2011 Census aggregate data., 2021) and therefore more research is needed to better examine the ethnic disparities in flu vaccine uptake. Thirdly, it should be noted that the current data reflects the decision-making processes of a population during a pandemic situation and thus, the profile of PAPM stages may have been unusually influenced by this. However, as we still in the aftermath of the pandemic and the flu with likely co-circulate with COVID and other infectious diseases, key lessons can still be learned that can promote vaccine acceptance for flu in future years. The strength of this study lies in the well-validated theoretical constructs on which the survey questionnaire was developed, and the large dataset of responses collected, which allowed for advanced statistical analyses, from a wide sample across all geographical regions in England.

## Conclusion

This is the first study to explore flu vaccination behaviour using a decision-making stage model which allows for a more nuanced approach along the continuum of vaccine hesitancy. It has provided insights about factors related to the decision of those who are in the ‘unengaged’ or ‘undecided’ stages of vaccination intention and hold beliefs that are more flexible and can change over time, as opposed to the ‘decided not to vaccinate’ group who have much more stable beliefs. This study was conducted with a group of participants who were 50 years of age or older and at a time when public health guidance strongly recommended the flu vaccine for this age group, particularly aiming to avoid co-occurrence with COVID-19, and was offered free of charge (or was covered by most employers). Yet we found that 17% of participants were in the ‘unengaged’ or ‘undecided’ stages. This highlights the need for targeted public health interventions aimed at increasing the uptake of flu vaccination and thus, creating a culture of flu vaccination. Our findings contribute to a better understanding of the key factors underlying each stage of the decision-making process in relation to the seasonal flu vaccine and can help inform policy recommendations for future immunisation programmes.

## Supplemental Material

sj-docx-1-hpq-10.1177_13591053241296650 – Supplemental material for Understanding the public’s decision-making about seasonal flu vaccination during a pandemic: Application of the precaution adoption process model Supplemental material, sj-docx-1-hpq-10.1177_13591053241296650 for Understanding the public’s decision-making about seasonal flu vaccination during a pandemic: Application of the precaution adoption process model by Carly Meyer, Vivi Antonopoulou, Louis Goffe, Aikaterini Grimani, Fiona Graham, Jan Lecouturier, Mei Yee Tang, Paul Chadwick and Falko F Sniehotta in Journal of Health Psychology
