# Supplementary material for: Understanding the public’s decision-making about seasonal flu vaccination during a pandemic: Application of the precaution adoption process model
Source: J Health Psychol. 2024 Nov 22;30(11):2984–3000. doi: 10.1177/13591053241296650 (PMC12433526; doi:10.1177/13591053241296650)
Supplement: sj-docx-1-hpq-10.1177_13591053241296650 – Supplemental material for Understanding the public’s decision-making about seasonal flu vaccination during a pandemic: Application of the precaution adoption process model [file sj-docx-1-hpq-10.1177_13591053241296650.docx]

**Supplementary files**

*Supplementary file 1.*  Sample characteristics (N = 2,004)

| **Variable** |  |  |
| --- | --- | --- |
| Age – M (SD) | 63.61 | (8.45) |
| Gender – N (%)  - Female  - Male  - Other | 1,022  981  1 | (51.00)  (48.95)  (0.05) |
| Region – N (%)  - East Midlands  - East of England  - London  - North East  - North West  - South East  - South West  - West Midlands  - Yorkshire and the Humber | 181  220  297  104  259  320  200  223  200 | (9.03)  (10.98)  (14.82)  (5.19)  (12.92)  (15.97)  (9.98)  (11.13)  (9.98) |
| Ethnicity – N (%)  - White British  - Not white British | 1,843  155 | (91.97)  (7.73) |
| Education status – N (%)  - No formal qualification  - High school qualification (e.g., BTEC, GCSE, A-levels)  - University diploma/degree  - Other qualification | 148  883  512  453 | (7.39)  (44.06)  (25.55)  (22.60) |
| Employment status – N (%)  - Employed  - Not employed | 751  1,245 | (37.48)  (62.13) |
| Household income – N (%)  - Less than £30,000  - More than £30,000  - Prefer not to say | 988  852  164 | (49.30)  (42.51)  (8.18) |
| Asked to shield – N (%)  - Yes  - No | 397  1,599 | (19.81)  (79.79) |
| Previous COVID-19 infection – N (%)  - Yes  - No | 216  1,786 | (10.78)  (89.12) |

*Supplementary file 2.* Overview of subscale items included in the analysis

| **Subscale and included items** | **Scale / response options** | **Cronbach’s alpha** |
| --- | --- | --- |
| **PAPM STAGE**   - Which of the following best describes your thoughts about having a seasonal flu vaccine this year? | “I’ve not yet thought about having the flu vaccine” (Stage 2); “I’m not yet sure about having the flu vaccine, but will probably (NOT) have it” (Stage 3); “I’ve decided I DON'T want to have the flu vaccine” (Stage 4); “I’ve decided I DO want to have the flu vaccine” (Stage 5); and “I’ve had the flu vaccine” (Stage 6) | n/a |
| **PREVIOUS FLU VACCINE EXPERIENCE** |  |  |
| **Previous flu vaccine experience** |  |  |
| - When was the last time you had your flu vaccine? | This year / last year / two years ago / more than two years ago / I have never had one / don’t know or can’t remember | n/a |
| - How often do you have the seasonal flu vaccine? | Rarely / some years / most years / every year | n/a |
|  |  |  |
| **Previous flu vaccine: Overall experience**   - Having the vaccine was ... *painful / tolerable* - I believe the vaccine was ... *worthless / valuable* - After having the vaccine, I experienced … *strong side effects / no side effects* - Overall, I found having the vaccine was … *a terrible experience / a good experience* | 5-point scale | 0.77 |
| **PSYCHOLOGICAL CONSTRUCTS** |  |  |
| **HEALTH BELIEF MODEL** |  |  |
| **Perceived severity**   - I believe I would be very sick if I caught the flu | 5-point scale (*strongly disagree – strongly agree*) | n/a |
| **Perceived susceptibility**   - I believe that I’m at high risk of catching the flu compared to others - I believe my immune system is strong enough to protect me against flu | 5-point scale (*strongly disagree – strongly agree*) | ^†^0.05 |
| **Perceived benefits**   - A flu vaccine will protect me against catching the flu - A flu vaccine would reduce the severity of a possible future flu infection - If I have a flu vaccine, I would be less likely to spread the flu to others - A mass flu vaccine programme will protect the vulnerable from catching the flu - A mass flu vaccine programme, will help protect the NHS | 5-point scale (*strongly disagree – strongly agree*) | 0.89 |
| **Perceived safety**   - I believe that the flu vaccine is very safe - I’m NOT concerned about the possible side effects of a flu vaccine | 5-point scale (*strongly disagree – strongly agree*) | 0.69 |
| **THEORY OF PLANNED BEHAVIOUR** |  |  |
| **Vaccine attitudes**   - Having a flu vaccine this year would be beneficial - Having a flu vaccine this year would be tolerable | 5-point scale (*strongly disagree – strongly agree*) | 0.88 |
| **Subjective norms**   - My family and friends have said they would get the flu vaccine once it's available to them - My family would expect me to have the flu vaccine - My GP would expect me to have the flu vaccine - ^‡^My employer would expect me to have the flu vaccine | 5-point scale (*strongly disagree – strongly agree*) | 0.81 (3 items) / 0.83 (4 items) |
| **Perceived control**   - I feel in total control as to whether I will have the flu vaccine | 5-point scale (*strongly disagree – strongly agree*) | n/a |
| **Anticipated regret**  How much would you regret that you did not have the flu vaccine if it was recommended you have one?   - You were hospitalised and admitted to the intensive care unit as a result of catching the flu - You caught the flu and passed it on to a friend - You caught the flu and passed it on to a family member | 5-point scale (*not at all – a great deal*) | 0.96 |
| **OTHER FACTORS** |  |  |
| **Vaccine knowledge**   - I know enough about how effective the flu vaccine is to make an informed decision about whether or not to get the vaccine - I know enough about how the flu vaccine will help reduce the spread of the virus - I know enough about the safety of a flu vaccine to make an informed decision about whether or not to get the flu vaccine | 5-point scale (*strongly disagree – strongly agree*) | 0.89 |
| **Trust**   - Information from the Government about the seasonal flu vaccine can be trusted | 5-point scale (*strongly disagree – strongly agree*) | n/a |
| **Fears of having a vaccine**   - In general, most reactions (or side effects) to flu vaccination do not last longer than two days - In general, there is much more risk of side-effects from COVID-19 vaccination vs. the regular seasonal flu vaccination - In general, all vaccinations can cause side-effects - I am scared of needles | Yes / no / don’t know | n/a |

*Note*. †each item represented separately in analyses due to low internal consistency; ‡only asked to people who reported they were employed

*Supplementary file 3.* Pairwise correlations between potential predictor variables (N = 2,004)

| Variables | (1) | (2) | (3) | (4) | (5) | (6) | (7) | (8) | (9) | (10) | (11) | (12) | (13) | (14) | (15) |
| --- | --- | --- | --- | --- | --- | --- | --- | --- | --- | --- | --- | --- | --- | --- | --- |
| (1) Age | 1.000 |  |  |  |  |  |  |  |  |  |  |  |  |  |  |
| (2) General health | 0.005 | 1.000 |  |  |  |  |  |  |  |  |  |  |  |  |  |
| (3) Previous vaccine experience | 0.137 | -0.059 | 1.000 |  |  |  |  |  |  |  |  |  |  |  |  |
| (4) Flu vaccine frequency | 0.313 | 0.118 | 0.214 | 1.000 |  |  |  |  |  |  |  |  |  |  |  |
| (5) Perceived severity | 0.138 | 0.197 | 0.101 | 0.370 | 1.000 |  |  |  |  |  |  |  |  |  |  |
| (6) Perceived susceptibility: high risk of catching flu | 0.080 | 0.254 | 0.100 | 0.358 | 0.568 | 1.000 |  |  |  |  |  |  |  |  |  |
| (7) Perceived susceptibility: immune system strong enough | 0.046 | -0.228 | 0.078 | -0.102 | -0.133 | -0.024 | 1.000 |  |  |  |  |  |  |  |  |
| (8) Perceived safety | 0.182 | -0.070 | 0.466 | 0.456 | 0.264 | 0.250 | 0.102 | 1.000 |  |  |  |  |  |  |  |
| (9) Vaccine attitudes | 0.223 | 0.017 | 0.508 | 0.576 | 0.412 | 0.342 | -0.076 | 0.719 | 1.000 |  |  |  |  |  |  |
| (10) Subjective norms | 0.324 | 0.041 | 0.320 | 0.524 | 0.465 | 0.430 | 0.027 | 0.580 | 0.701 | 1.000 |  |  |  |  |  |
| (11) Perceived control | 0.243 | -0.017 | 0.306 | 0.322 | 0.208 | 0.146 | 0.068 | 0.554 | 0.538 | 0.460 | 1.000 |  |  |  |  |
| (12) Vaccine knowledge | 0.236 | -0.053 | 0.388 | 0.463 | 0.328 | 0.310 | 0.075 | 0.691 | 0.673 | 0.641 | 0.602 | 1.000 |  |  |  |
| (13) Trust | 0.164 | -0.070 | 0.313 | 0.347 | 0.278 | 0.233 | 0.098 | 0.584 | 0.569 | 0.532 | 0.432 | 0.615 | 1.000 |  |  |
| (14) Anticipated regret | 0.157 | 0.046 | 0.160 | 0.303 | 0.243 | 0.128 | -0.162 | 0.274 | 0.454 | 0.367 | 0.263 | 0.305 | 0.278 | 1.000 |  |
| (15) Perceived benefits | 0.152 | -0.023 | 0.353 | 0.402 | 0.352 | 0.293 | 0.050 | 0.548 | 0.641 | 0.571 | 0.394 | 0.570 | 0.545 | 0.405 | 1.000 |
|  | | | | | | | | | | | | | | | |
